# Supplementary material for: Potential Therapeutic Effects of Thiazolidinedione on Malignant Glioma
Source: Int J Mol Sci. 2022 Nov 4;23(21):13510. doi: 10.3390/ijms232113510 (PMC9657575; doi:10.3390/ijms232113510)
Supplement: Supplementary file 1 [file ijms-23-13510-s001.zip › ijms-1907105-supplementary.pdf]

Supplementary Data

A

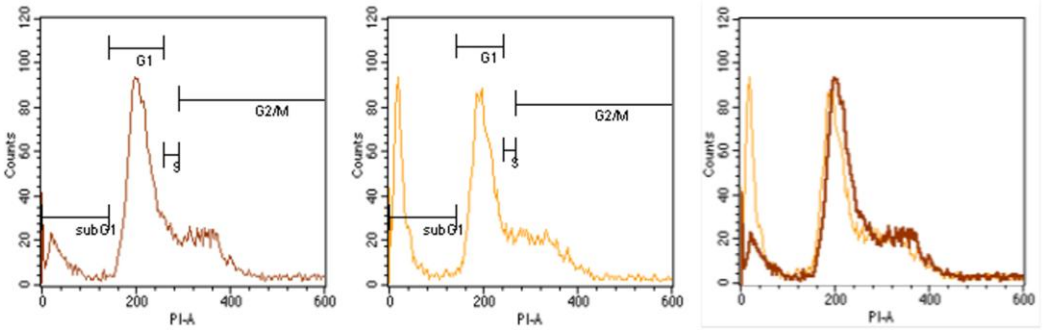

B

Control

Marker	% Gated
All	100.00
subG1	10.49
G1	52.20
S	7.10
G2/M	23.13

Troglitazone (20 μM)

Marker	% Gated
All	100.00
subG1	24.83*
G1	43.26
S	5.05
G2/M	24.33

D

Control

Marker	% Gated
All	100.00
subG1	7.52
G1	53.38
S	7.84
G2/M	24.90

15d-PGJ2 (10 μM)

Marker	% Gated
All	100.00
subG1	23.41*
G1	39.13
S	7.07
G2/M	25.09

C

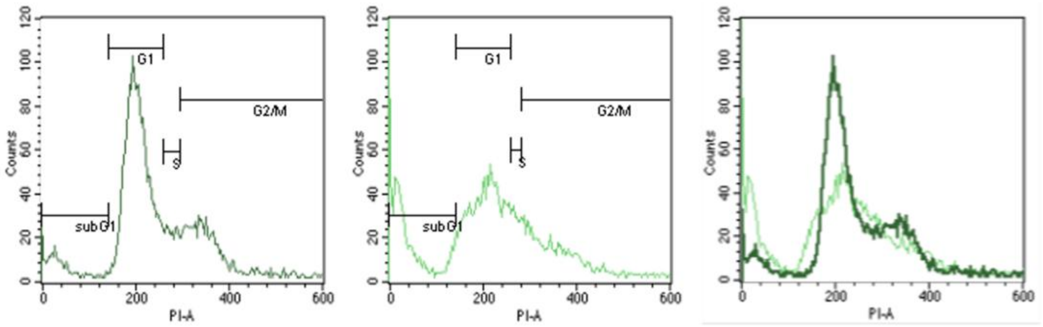

Supplementary Figure S1: Detection of U87 cells death treated with thiazolidinedione analogs (Troglitazone and 15d-PGJ2) by Flow Cytometry
(A) Cell cycle histogram showing phase distribution (subG0, G1, S and G2/M) of cells at 24 hours post Troglitazone -induction in U87 cells. Quantification for % gated population. (B) Left panel for control, and Right panel for Troglitazone treatment. (C) Cell cycle histogram showing phase distribution (subG0, G1, S and G2/M) of cells at 24 hours post 15d-PGJ2-induction in U87 cells. Quantification for % gated population. (D) Left panel for control, and Right panel for 15d-PGJ2 treatment. Data are expressed as mean ± SD (n = 4) *p < 0.05 vs. control group cells.

Supplementary Table S1: Primary and secondary antibodies for western blot

Antibody	Catalog No.	Dilution	Brand	Headquarter
α-tublin	sc-8035	1:500	Santa Cruz	Burlington, MA, USA
Calpain I	sc-7531	1:500	Santa Cruz	Burlington, MA, USA
Calpain II	sc-7533	1:1000	Santa Cruz	Burlington, MA, USA
CDK2	sc-163	1:1000	Santa Cruz	Burlington, MA, USA
CDK4	sc-601	1:1000	Santa Cruz	Burlington, MA, USA
Cyclin D1	sc-717	1:500,	Santa Cruz	Burlington, MA, USA
Cyclin E	sc-481	1:1000	Santa Cruz	Burlington, MA, USA
LAMP 1	ab25630	1:500	Abcam	Cambridge, United Kingdom
p-STAT3(Tyr705)	#9131	1:500	Cell Signaling	Danvers, Massachusetts ,USA
SHP-1	sc-7533	1:500	Santa Cruz	Burlington, MA, USA
SHP-2	sc-7533	1:500	Santa Cruz	Burlington, MA, USA
Ubiquitin	sc-7533	1:1000	Santa Cruz	Burlington, MA, USA
LC3B I/II	ab51520	1:1000	Abcam	Cambridge, UK.
Goat anti-mouse HRP	sc-2005	1:200	Santa Cruz	Burlington, MA, USA
Goat anti-rabbit HRP	sc-2004	1:200	Santa Cruz	Burlington, MA, USA
Goat anti-rabbit Rhodamine	03-15-06	1:200	Kirkegaard & Perry Laboratories, Inc	Gaithersburg, Maryland, USA
Goat anti-mouse FITC	172-1806	1:200	Kirkegaard & Perry Laboratories, Inc	Gaithersburg, Maryland, USA
Goat anti-rabbit FITC	02-15-16	1:200	Kirkegaard & Perry Laboratories, Inc	Gaithersburg, Maryland, USA
Donkey anti-goat HRP	sc-2020	1:200	Santa Cruz	Burlington, MA, USA
